# Phosphatidylcholine Liposomes Reprogram Macrophages toward an Inflammatory Phenotype

**DOI:** 10.3390/membranes13020141

**Published:** 2023-01-21

**Authors:** David M. Cauvi, Dennis Hawisher, Julia Derunes, Antonio De Maio

**Affiliations:** Department of Surgery, School of Medicine, University of California San Diego, La Jolla, CA 92093, USA

**Keywords:** phospholipids, inflammation, cytokines/chemokines

## Abstract

Phospholipids are the major components of cellular membranes and cell-derived vesicles such as exosomes. They are also key components of artificial lipid nanoparticles, allowing the encapsulation and transport of various biological or chemical cargos. Both artificial and natural vesicles could be captured by cells delivering important information that could modulate cellular functions. However, the potential contribution of phospholipids within vesicles altering cellular physiology has been largely underestimated. Here, we showed that macrophages exposed to liposomes made exclusively with palmitoyl oleoyl phosphatidylcholine (POPC) in vivo resulted in a dramatic alteration of the transcriptome profile. Differential gene expression analysis indicated that the exposure to POPC liposomes resulted in a change in the expression of 1598 genes. Moreover, 146 genes were upregulated, and 69 genes were downregulated by incubation with POPC liposomes in contrast to palmitoyl oleoyl phosphatidylserine (POPS) exposure. Signaling pathway impact analysis revealed that 24 signaling pathways were significantly modulated after exposure to POPC liposomes, including the activation of the NF-κB pathway. Indeed, the expression of several cytokines (TNF-α, IL-6, and IL-10) and chemokines (Cxcl1 and Cxcl2) were increased. These observations were validated by the exposure of macrophages to POPC liposomes in culture conditions. In addition, the proteomic analysis of peritoneal cells exposed to POPC liposomes performed by mass spectrometry revealed that the expression of 107 proteins was downregulated after POPC exposure, whereas the expression of 12 proteins was significantly upregulated by this treatment, including seven proteins involved in the neutrophil degranulation pathway. This observation was confirmed by flow cytometry analysis showing the rapid recruitment of neutrophils into the peritoneal cavity after POPC exposure. Overall, these findings demonstrate that the presence of phospholipids within artificial and natural vesicles could be responsible for changes in the function of target cells.

## 1. Introduction

Lipid nanoparticles (LNP) have emerged as ideal carriers for the delivery of genetic drugs based on nucleic acids directed at gene editing, silencing, or protein expression [1,2]. In addition, LNP have been used for the development of vaccines, which has resulted in the great success against severe acute respiratory syndrome coronavirus 2 (SARS-CoV-2), where the viral spike protein mRNA was encapsulated into the particles [3,4,5,6]. The key advantage of the use of nucleic acid encapsulated LNP is that the DNA or RNA cargo is protected from the activity of circulating degrading enzymes present in body fluids. The main scaffold components of LNP are phospholipids that, due to their amphiphilic characteristics, assemble into lipid bilayers forming vesicles by a spontaneous process, entropically driven [7]. This phospholipid membrane acts as a barrier between the vesicle lumen and the external environment protecting their internal cargo. In some instances, particularly for use as vaccines, phospholipid nanoparticles contain cholesterol to increase the stability of the vesicle and are decorated with polyethylene glycol for integrity preservation [3].

Loading nucleic acids into LNP is not a trivial process. The addition of the potential cargo during the formation of the liposomes only resulted in equilibrium with the external substrate concentration, which is not very efficient for therapeutic goals. A great advance was the introduction of an ionizable phospholipid that is positively charged at low pHs and neutral at physiological pH conditions. Thus, the nucleic acids are loaded into liposomes at low pH, increasing the internal concentration of the substrate [2,8]. In spite of this advancement, the next step is how to direct LNP to specific target cells. In this regard, the incorporation of surface molecules on the vesicles has been shown to direct the particles to specific cell types, such as hepatocytes [1]. However, a major challenge for the in vivo delivery of LNP is to avoid their capture by macrophages due to the higher capacity of these cells to ingest foreign particles. Another major question that remains to be answered concerns the mechanisms of LNP uptake by cells, the intracellular fate of the vesicles, and the transport of the nucleic acid cargo into the cytosol to carry out its biological activity.

As indicated above, the main components of LNP are phospholipids, particularly phosphatidylcholine, which has been coined the helper phospholipid to differentiate from other phospholipid components. In this regard, phospholipids have been considered as inert elements [9]. However, it could be argued that these natural molecules could play an independent role in modulating cellular responses [10]. Indeed, we have reported that liposomes made exclusively with palmitoyl oleoyl phosphatidylserine (POPS) induced robust activation of the innate immune response in peritoneal cells in vivo [11]. In this report, we expand our prior observations by presenting data demonstrating the potential effect of phosphatidylcholine modulating the inflammatory response by macrophages, both in vivo and ex vivo models.

## 2. Materials and Methods

### 2.1. Animals

CD-1 (Crl:CD1 (ICR)) mice were acquired from Charles River and maintained under specific, pathogen–free conditions at the University of California, San Diego Animal Facility (La Jolla, CA, USA). Experiments were performed on 9-week-old male mice. All animal procedures were approved by the UCSD Institutional Animal Care and Use Committee, protocol number: S07177.

### 2.2. Cell Isolation and Culture

Peritoneal cells were collected by lavage. Briefly, 5 mL of serum-free phenol-red free RPMI1640 were injected into the peritoneal cavity of CD-1 mice, and after gentle massage of the peritoneum to dislodge any loosely attached cells, fluid was collected. Cell suspensions were immediately centrifuged for 10 min at 350× *g* and resuspended in PBS without Ca^2+^/Mg^2+^ supplemented with 0.5% BSA for counting.

J774A.1 macrophages. Cells were obtained from ATCC (TIB-67, ATCC, Manassas, VA, USA) and maintained in RPMI1640 with L-glutamine and penicillin/streptomycin and supplemented with 10% FBS.

Bone marrow cells were obtained from the femur and tibia of male CD-1 mice (9 weeks old). The bone marrow was flushed out with cold PBS supplemented with 2% heat-inactivated FBS using a 22-gauge needle. Single-cell suspensions were collected after passing through a 22-gauge needle 4–6 times to dissociate cell clumps. Cells were then passed through a 70-µm cell strainer to eliminate residual clumps and centrifuged at 500× *g* for 8 min at 4 °C. Cells were treated with red blood cell lysis buffer (eBioscience, San Diego, CA, USA), washed, and centrifuged as described above. Isolated bone marrow cells were resuspended (2 × 10^6^ cells/mL) in RPMI1640 with L-glutamine and penicillin/streptomycin and supplemented with 10% FBS and 10 ng/mL recombinant M-CSF (R&D Systems, Minneapolis, MN, USA). The culture medium was changed after 3 days, and non-adherent cells were discarded. On day 7, the presence of mature bone marrow-derived Mϕ was evaluated by expression of CD11b and F4/80 using fluorophore-conjugated antibodies and flow cytometry. On average, more than 98% of the cells were CD11b^+^ and F4/80^+^, corresponding to Mϕ.

Resident naïve peritoneal macrophages were isolated by peritoneal cavity lavage as described above. Cell suspensions were immediately centrifuged for 10 min at 350× *g*, resuspended in RPMI1640 with L-glutamine and penicillin/streptomycin and supplemented with 10% FBS, and seeded on a 12-well plate at a concentration of 5 × 10^5^ cells per well. Cells were then allowed to attach for 1 h at 37 °C in a CO_2_ incubator. Non-adherent cells were removed, and fresh medium was added. Cells were incubated for an additional 16 h, cellular viability was determined by the Trypan-blue exclusion test (90% viability was considered acceptable), and purity of the cell population was determined by flow cytometry using F4/80 antibodies. A 90–95% population of macrophages was considered acceptable.

### 2.3. Liposome Preparation and Treatment

1-palmitoyl-2-oleoyl-*sn*-glycero-3-phosphocholine (POPC) in chloroform (Avanti Polar Lipids, Alabaster, AL, USA) was dried under a nitrogen stream. Liposomes were formed by rehydrating the dried lipid film (400 μg) in 50 mM endotoxin-free Tris Buffer pH 7.4 (120 μL) and vortexing every 5 min for 30 min. In POPC liposome uptake experiments, liposomes were formed by rehydrating the dried lipid film (400 μg) in 50 mM endotoxin-free Tris Buffer pH 7.4 (120 µL) containing either 0.01 mg/mL AlexaFluor647-labeled dextran (10,000 MW; Invitrogen, Carlsbad, CA, USA) or 0.5 mg/mL AlexaFluor488-labeled dextran (10,000 MW; Invitrogen) and vortexing every 5 min for 30 min. The preparation was extruded by 15 passages through a 100 nm membrane filter using a mini-extruder apparatus (Avanti Polar Lipids). Liposomes were then centrifuged at 100,000× *g* for 60 min at 4 °C and resuspended in sterile PBS at a concentration of 1 mg/mL. The size distribution and concentration for each liposome preparation were verified by Nanoparticle Tracking Analysis (NTA, Nanosight NS300, Malvern Panalytical, Malvern, UK). The level of endotoxin contamination was determined using the ToxinSensor™ *Chromogenic LAL* Endotoxin Assay Kit (GenScript). The concentration of endotoxin found in POPC liposome preparation was generally less than 0.01 EU/mL, which corresponds to less than 0.002 ng/mL of endotoxin contamination.

### 2.4. RNA Extraction, cDNA Production and Quantitative Real-Time PCR (qPCR)

Levels of mRNA were measured by quantitative real-time PCR (qPCR). Peritoneal cells and bone marrow-derived macrophages were resuspended in TRIzol reagent following treatment and mixed thoroughly. RNA was purified according to the manufacturer’s protocol and treated with DNase I (DNA-free kit, Ambion, Austin, TX, USA) to remove any DNA contamination. DNA-free RNA was then reverse transcribed to cDNA using the High Capacity Reverse Transcription Kit (Applied Biosystems, Foster City, CA, USA). Newly synthesized cDNA was further diluted and stored at −20 °C. The cDNA levels of genes were measured by quantitative real-time PCR (qPCR) using the QuantiTect SYBR Green PCR kit (Qiagen, Valencia, CA, USA) with QuantiTect validated primer sets (Tnfa: QT00104006; Il6: QT00098875; Il10: QT00106169; Cxcl1 (KC): QT00115647; Cxcl2 (MIP2-α): QT00113253, all from Qiagen). All PCR reactions were performed using the StepOnePlus Real-Time PCR System (Applied Biosystems). Melting curve analysis was performed for each primer set to ensure amplification specificity. Corresponding standard curves were added in each PCR reaction. The housekeeping gene GAPDH (QT01658692, Qiagen, Valencia, CA, USA) was used to normalize data to cDNA inputs. The results are expressed as copy numbers of the target gene per copy numbers of GAPDH.

### 2.5. Immunoblot Analysis

J774 cells treated with PBS (vehicle control), LPS (100 ng/mL, 10 min.), or POPC (10, 20, 40, and 60 min.) were lysed in the RIPA lysis buffer containing 0.01M sodium fluoride as serine/threonine phosphatases inhibitor, vortexed for 30 s and cell homogenates were incubated for 30 min at 4 °C. Samples were then centrifuged at 10,000× *g* for 8 min at 4 °C. The supernatant was collected, and a 10 µL aliquot was used to determine protein concentration using a BCA protein assay (Pierce Biotechnology, Rockford, IL, USA). Cell homogenates were mixed with NuPAGE LDS sample buffer (Life Technologies, Carlsbad, CA, USA), and 60 µg of total protein was resolved by SDS-PAGE using NuPAGE 4–12% Bis-Tris gels (Life Technologies, Carlsbad, CA). Proteins were then transferred to nitrocellulose membranes and blocked with 5% BSA diluted in Tris-buffered saline (TBS) for 1 h at 23 °C. Blots were probed with a rabbit anti-phospho-NF-kB p65 mAb (1/2000, clone 93H1; Cell Signaling Technology, Danvers, MA, USA) in 5% BSA-TBS and incubated overnight at 4 °C followed by three 15 min washes with TBS supplemented with 0.1% Tween 20 (TBST) at 23 °C. Blots were then incubated with HRP-conjugated goat anti-rabbit IgG antibodies (1:3000—Santa Cruz Biotechnology, Dallas, TX, USA) in 5% BSA-TBS for 1 h at 23 °C. After three 15 min washes in TBST, bands were detected by chemiluminescence using SuperSignal reagents (Pierce Biotechnology). As a loading control, blots were probed with mouse anti- β-actin monoclonal antibodies (1:3000—Thermo Scientific, Waltham, MA, USA) in 5% BSA-TBS for 16 h at 4 °C. Goat anti-mouse HRP-conjugated IgG secondary antibody (1:3000—Thermo Scientific) was used for 1 h at 23 °C, followed by chemiluminescence detection. Chemiluminescence data were acquired using the ChemiDoc™ MP system (Bio-Rad, Hercules, CA, USA) and densitometry analyses were performed using the Image Lab™ software (Bio-Rad).

### 2.6. Global Gene Analysis by RNA Sequencing

CD-1 mice were injected i.p. with 400 µg of POPC liposomes, and total peritoneal cells were then collected 1 h later by peritoneal lavage and processed for global gene analysis by RNA sequencing. Control animals received an equal volume of PBS. RNA was isolated using the RNeasy Mini Kit according to the manufacturer’s protocol (Qiagen, Valencia, CA, USA), and RNA integrity numbers (RIN) were determined by the Agilent 4200 Tapestation. RNA sequencing libraries were then generated from 1 µg of RNA using the TruSeq^®^ Stranded Total RNA Library Prep Gold Sample Prep Kit following the manufacturer’s instructions (Illumina, San Diego, CA, USA), modifying the shear time to 5 min. RNA libraries were multiplexed and sequenced with 75 base pair (bp) single reads (SR75) to a depth of approximately 40 million reads per sample on an Illumina HiSeq4000 (Illumina, San Diego, CA, USA). Quality control of the raw fastq files was performed using the FastQC software tool. Sequencing reads were aligned to the mouse genome (mm10) using the STAR v2.5.1a aligner. Read quantification was performed with RSEM v1.3.0 and GENCODE annotation (Mus_musculus.GRCm38.68.gtf). The R BioConductor packages edgeR and limma were used to implement the Limma-voom method for differential expression analysis. The experimental design was modeled upon treatment (~0 +treatment). Significance was defined by using an adjusted *p*-value cut-off of 0.05 after multiple testing corrections using a moderated t-statistic in Limma. Functional enrichment of the differentially expressed genes was performed using the Bioconductor GSVA package for implementing GSEA. The Signaling Pathway Impact Analysis (SPIA) and overrepresentation analysis were performed by using the Bioconductor package and the WebGestalt toolkit, respectively. The data have been deposited in NCBI’s Gene Expression Omnibus and are accessible through GEO series accession number GSE115489.

### 2.7. Flow Cytometry Analysis of Peritoneal Cells

Peritoneal cells were collected by lavage of the peritoneum as described above. Cells were centrifuged for 10 min at 300× *g*, resuspended in PBS without Ca^2+^/Mg^2+^ supplemented with 0.5% BSA (FACS staining buffer, FSB), and counted. Peritoneal cells (5 × 10^5^ cells/tube) were then incubated for 15 min with 0.5 µg of FcγR blocking antibodies (Fc block; BD Biosciences, San Jose, CA, USA), followed by antibody staining for 30 min in the dark at 4 °C. Cells were then washed, centrifuged, and resuspended in FSB for analysis. Each anti-mouse antibody was added at 0.5 µg/tube and included FITC-conjugated anti-Ly6G (clone 1A8, Biolegend, San Diego, CA, USA), PE-conjugated anti-CD11b (clone M1/70, eBioscience), PerCP-conjugated anti-CD19 (clone 1D3, BD Bioscience), APC-conjugated anti-F4/80 (clone BM8, eBioscience). Propidium iodide was also used to assess cell viability. Flow cytometry was performed using a FACSCanto II flow cytometer with FACSDiva software (BD Biosciences, San Jose, CA, USA). The data were analyzed using FlowJo software v.10.1 (Tree Star, Ashland, OR, USA).

### 2.8. POPC Liposome Uptake

For flow cytometry analysis, J774A.1 macrophages were incubated for different periods of time with POPC liposomes loaded with AlexaFuor647-labeled dextran. Cells were then collected by scraping and processed for flow cytometry analysis. For microscopy analysis, J774A.1 macrophages were plated onto Nunc Lab-Tek II Chamber slides (ThermoFisher Scientific, Waltham, MA, USA) and incubated for different periods of time with POPC liposomes loaded with AlexaFuor488-labeled dextran. Cells were then washed and fixed with 4% paraformaldehyde and the slides were mounted with Vectashield mounting medium containing DAPI (Vector Laboratories, Burlingame, CA). Fluorescence was acquired using an inverted fluorescence microscope (100× oil immersion objective, Eclipse TE300, Nikon Instruments, Melville, NY, USA) equipped with an AxioCam HRm (Zeiss, Pleasanton, CA, USA). Image analysis was performed using the Zen microscope software (Zeiss).

### 2.9. Mass Spectrometry Analysis

CD-1 mice were injected i.p. with 400 µg of POPC liposomes and total peritoneal cells were collected 1 h later by peritoneal lavage, centrifuged, and cell pellets were processed for mass spectrometry analysis by the Biomolecular and Proteomics Mass Spectrometry Facility (University of California, San Diego). Control animals received an equal volume of PBS. Briefly, samples were digested with trypsin and analyzed by HPLC coupled with tandem mass spectrometry (LC-MS/MS) using nano-spray ionization (TripleTOF 5600 hybrid mass spectrometer (AB SCIEX)). MS/MS spectra acquisition, peptide/protein identification, quantification of the data, and statistical calculations were performed using the peptide-feature-based PEAKS Studio X (Bioinformatics Solutions Inc., Canada) [12].

### 2.10. Statistical Analysis

All data were analyzed using GraphPad Prism software (GraphPad Prism Software, San Diego, CA, USA). Significance was analyzed using one-way ANOVA followed by Tukey’s Multiple Comparison Test, two-way ANOVA followed by Bonferroni post-tests, or unpaired Student’s *t*-test. A *p* < 0.05 value was considered statistically significant.

## 3. Results

### 3.1. POPC Liposomes Trigger a Robust Inflammatory Response in Macrophages

**Figure 1:**
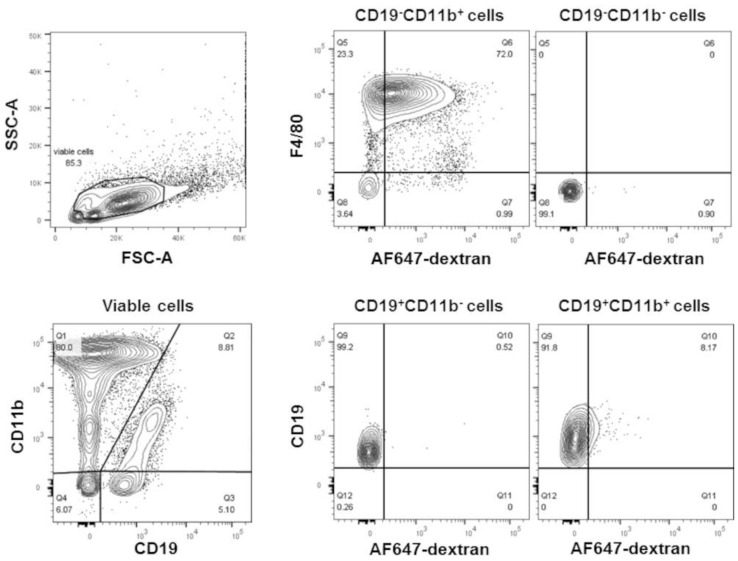
POPC liposomes are readily captured by peritoneal macrophages. POPC liposomes (400 µg) loaded with AlexaFluor647-labeled dextran were injected (1 mL in PBS) into the peritoneal cavity of CD-1 mice. After 1 h, peritoneal cells were collected by lavage, centrifuged, counted, and resuspended in FACS staining buffer. After blocking Fc receptors with anti-FcγR antibodies, peritoneal cells were incubated with FITC-conjugated anti-F4/80, PE-conjugated anti-CD11b, and PerCP-conjugated anti-CD19. Propidium iodide was also used to determine cell viability. Flow cytometry was performed using a FACSCanto II flow cytometer with FACSDiva software. The data were analyzed using FlowJo software. Peritoneal macrophages are defined as CD19^−^CD11b^+^F4/80^+^; B cells are defined as CD19^+^CD11b^−^, and B1 cells are defined as CD19^+^CD11b^+^.

We investigated whether phosphatidylcholine within liposomes altered cellular gene expression. Male CD-1 mice were injected into the peritoneal cavity with liposomes (100 nm in size) made exclusively of POPC (400 µg, corresponding to approximately 2 × 10^12^ particles). The liposomes were labeled by incorporating AlexaFluor647-conjugated dextran (10 kD) into the vesicle lumen. As a control, an equal volume of PBS (carrier) was injected into the peritoneal cavity and used to determine the baseline. Peritoneal cells were collected by lavage after 1 h of the injection to avert secondary effects due to the potential systemic distribution of the particles. Over 70% of the liposomes were captured by peritoneal macrophages (CD19^−^CD11b^+^F4/80^+^), and a very small number (about 8%) were also found in B1 cells (CD19^+^CD11b^+^) (Figure 1).

**Figure 2:**
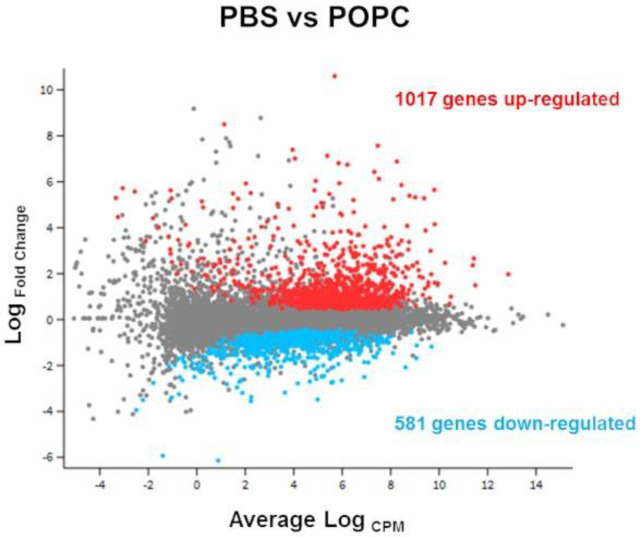
POPC liposomes significantly modify peritoneal cell transcriptome. CD-1 mice (*n* = 3 per group) were injected with POPC liposomes (400 µg) or an equal volume of PBS (carrier). Peritoneal cells were collected 1 h after the injection and processed for RNA isolation and RNA-seq analysis. The Glimma plot showing the differential gene expression between cells treated with POPC liposomes and PBS is presented, displaying the log-fold-change vs. average expression for each gene. Genes up-regulated in POPC liposomes vs. PBS are shown as red dots, while genes that are down-regulated in POPC liposomes vs. PBS are depicted as blue dots. A total of 1017 genes were up-regulated by POPC treatment, whereas 581 genes were down-regulated after POPC exposure.

In order to address whether there was a change in the transcriptome of the liposome- capturing cells, the experiment was repeated using non-labeled POPC liposomes, RNA was extracted from the peritoneal cells, and changes in the total transcriptome were determined by next-generation sequencing (RNA-seq). A total of 12,760 genes were detected by this method. Differential gene expression analysis showed that the exposure to POPC liposome resulted in a change in the expression of 1598 genes in comparison with PBS (Figure 2).

**Figure 3:**
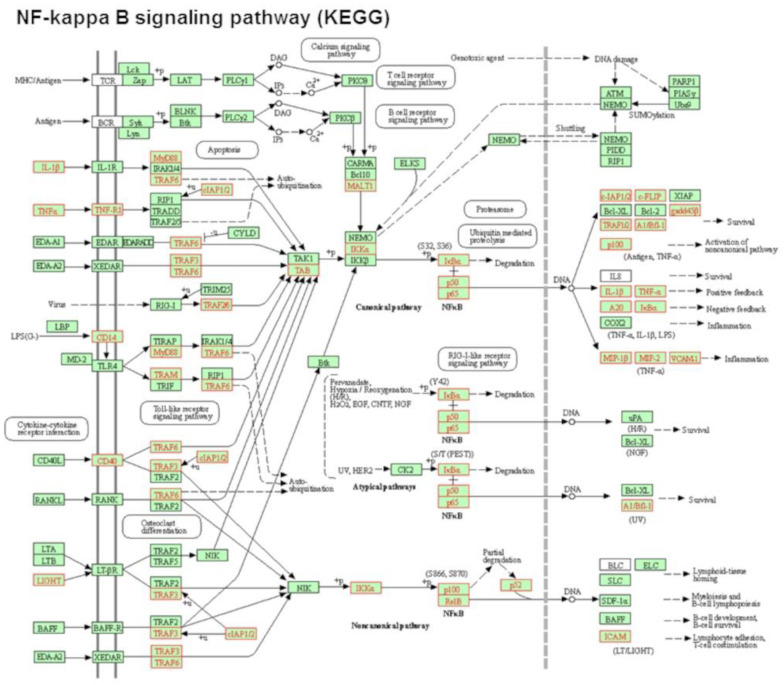
POPC liposomes induce the activation of the NF-kappa B signaling pathway. Analysis of the most significantly activated KEGG pathway by POPC liposome treatment corresponding to the NF-kappa B signaling pathway. Genes that are up-regulated in this pathway by POPC liposome exposure vs. PBS treatment are marked in red (29 genes). Statistical Pathway Impact Analysis (SPIA) shows a False Discovery Rate with adjusted global *p*-values (pGFdr) of 2.81 × 10^−09^ for this pathway.

Signaling pathway impact analysis (SPIA) revealed that 24 signaling pathways were significantly activated or inhibited after exposure to POPC liposomes (pGFdr < 0.2; Table 1). Among these pathways, activation of the KEGG NF-κB pathway, in which 29 genes were upregulated after POPC liposome treatment, showed the highest statistical significance (pGFdr = 2.81 × 10^−9^; Figure 3).

**Figure 4:**
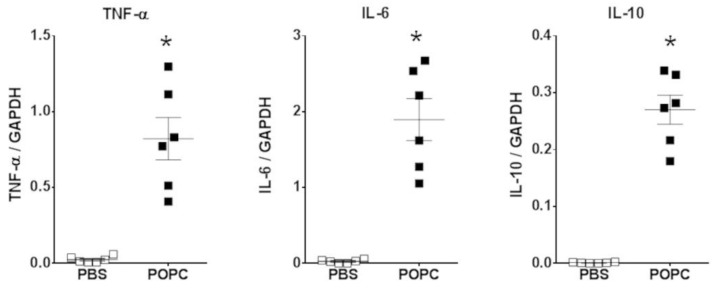
POPC liposome treatment increases the expression of inflammatory mediators in peritoneal cells. CD-1 mice (*n* = 6 per group) were injected with POPC liposomes (400 µg) or an equal volume of PBS (carrier). Peritoneal cells were harvested 1 h after the injection and processed for RNA isolation and qPCR analysis. Graphs show the mRNA levels for TNF-α, IL-6 and IL-10. The housekeeping gene GAPDH was used to normalize data to cDNA inputs. Results are expressed as means ± SEM, and statistical analysis for the comparison between groups was performed using unpaired Student’s *t*-test with * indicating *p* < 0.05.

These data strongly suggest that POPC liposomes activated the inflammatory response in peritoneal cells. Several inflammatory genes significantly upregulated by POPC exposure are thus presented in Table 2, including cytokines and chemokines. As proof of the rigor of our approach, the expression of the TNF-α, IL-6 and IL-10 in peritoneal cells was also analyzed by qPCR, showing a good correlation with RNAseq data (Figure 4). In addition to genes involved in the inflammatory process, the expression of other genes was regulated by incubation with POPC liposomes in comparison to the exposure to POPS liposomes as we have previously reported [11], including *Mfsd2a*, *Tril*, *Tnfsf4* that were activated (Table S1) and *Epha2*, *Tmem86a*, *Cln8* that were down-regulated (Table S2).

**Figure 5:**
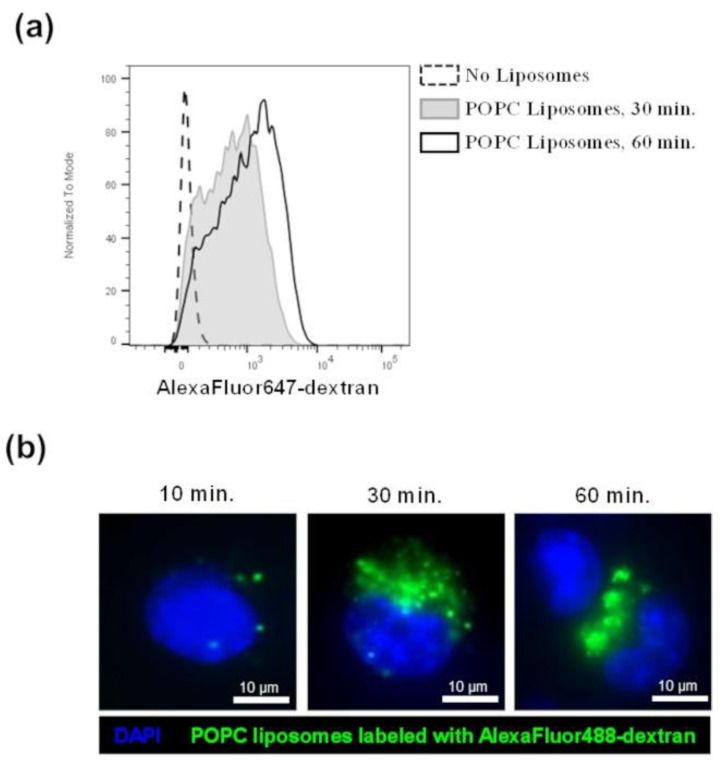
POPC liposomes are captured by J774A.1 macrophages. (**a**) J774A.1 cells were incubated with POPC liposomes (400 µg) loaded with AlexaFluor647-labeled dextran for either 30 or 60 min. Control cells received PBS vehicle only (no liposome). After incubation, cells were washed twice with FACS staining buffer and fluorescence was analyzed by flow cytometry. (**b**) J774A.1 cells were incubated with POPC liposomes (400 µg) loaded with AlexaFluor488-labeled dextran for either 15, 30, or 60 min. After incubation, cells were washed with PBS, fixed with 4% PFA, and washed again with PBS. Cells were then mounted with Vectashield Antifade Mounting medium containing DAPI for counterstaining. Images were acquired using a Nikon Eclipse TE300 fluorescent microscope equipped with the Zeiss AxioCam HRm camera and analyzed with the Zen 2.6 software.

To confirm that macrophages are indeed largely responsible for the inflammatory response observed after POPC stimulation in vivo, we incubated macrophages in culture conditions with POPC liposomes and measured the expression of TNF-α by qPCR as a readout of NF-kB activation. First, we verified that POPC liposomes loaded with fluorescent-labeled dextran were actively captured by J774A.1 macrophages by using flow cytometry (Figure 5a) or fluorescent microscopy (Figure 5b). The captured particles by macrophage corresponded to approximately 0.5 × 10^6^ POPC liposomes per Mϕ. Assuming that the content of lipid moieties per POPC liposome (100 nm in size) is about 1 × 10^5^ molecules, the increase of phospholipids per cell is about 10^10^ molecules.

**Figure 6:**
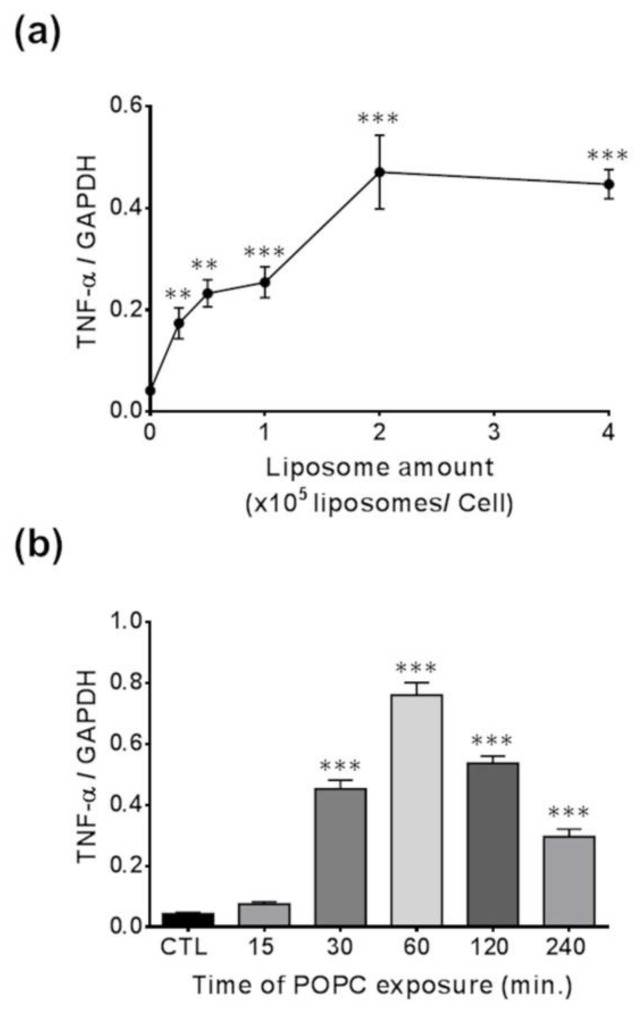
Dose- and time-response effect of POPC on TNF-α expression. (**a**) J774A.1 cells were treated with different ratios of POPC liposomes per cell for 1 h. Control cells received PBS vehicle only (no liposome). TNF-α mRNA levels were analyzed for each POPC liposome amount by qPCR. The housekeeping gene GAPDH was used to normalize data to cDNA inputs. Results (*n* = 4) are expressed as means ± SEM, and statistical analysis was performed using one-way ANOVA (*p* < 0.001) followed by Tukey’s Multiple Comparison Test with ** indicating *p* < 0.01 and *** indicating *p* < 0.001 compared to controls. (**b**) J774A.1 cells were treated with POPC liposomes (2 × 10^5^ liposomes/cell) for different time intervals, and TNF-α mRNA levels were analyzed by qPCR at each time point. The housekeeping gene GAPDH was used to normalize data to cDNA inputs. Results (*n* = 4) are expressed as means ± SEM and statistical analysis was performed using one-way ANOVA (*p* < 0.001) followed by Tukey’s Multiple Comparison Test with *** indicating *p* < 0.001 compared to controls.

We incubated J774A.1 macrophages with different concentrations of POPC liposomes for 1 h and found that the expression of TNF-α was significantly increased when 0.25 × 10^5^ POPC liposomes were added per cell as compared to PBS-treated controls, reaching a plateau after that concentration of particles (Figure 6a). Next, we exposed J774A.1 macrophages to POPC liposomes (4 × 10^5^ POPC liposomes per cell) for various periods of time and assessed changes in TNF-α expression. We observed a statistically significant increase in TNF-α gene expression after 30 min of POPC exposure as compared to PBS-treated controls, reaching maximum expression after 60 min of incubation with POPC liposome (Figure 6b).

**Figure 7:**
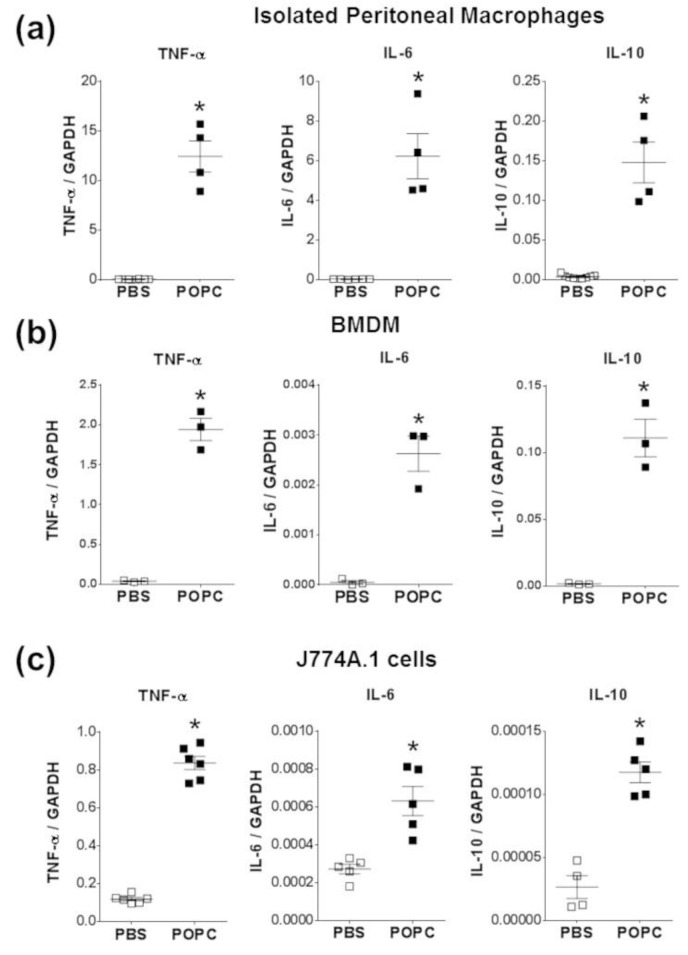
POPC liposomes trigger an inflammatory response in macrophages. (**a**) Resident naïve peritoneal macrophages were obtained from peritoneal cavity lavage as described in the Section 2. Briefly, peritoneal cells were allowed to attach for 1 h at 37 °C in a CO_2_ incubator. Non-adherent cells were removed, and fresh medium was added. Cells were then incubated for an additional 16 h, and the presence of macrophage markers CD11b and F4/80 was evaluated by flow cytometry. (**b**) Bone marrow-derived macrophages (Mϕ) were obtained from the femur and tibia of male CD-1 mice (9 weeks old). After isolation, bone marrow cells were depleted of red blood cells and resuspended (2 × 10^6^ cells/mL) in a culture medium supplemented with 10 ng/mL recombinant M-CSF. The culture medium was changed after 3 days, and non-adherent cells were discarded. The presence of mature bone marrow-derived Mϕ was evaluated by expression of CD11b and F4/80 and stimulated on day 7. (**c**) The macrophage cell line J774A.1 was obtained from ATCC and maintained in RPMI1640 with L-glutamine and penicillin/streptomycin and supplemented with 10% FBS. All macrophage types were exposed to POPC liposomes (2 × 10^5^ liposomes per cell) for 1 h. Levels of TNF-α, IL-6, and IL-10 mRNA were determined by qPCR with the housekeeping gene GAPDH used to normalize data to cDNA inputs. Results are expressed as means ± SEM, and statistical analysis for the comparison between groups was performed using unpaired Student’s *t*-test with * indicating *p* < 0.05.

To corroborate these observations made on cell lines, we also incubated peritoneal macrophages and bone marrow-derived macrophages (BMDMs) with POPC liposomes (4 × 10^5^ POPC liposomes per cell) for 1 h and measured the levels of TNF-α, IL-6 and IL-10 by qPCR. As we observed in peritoneal cells (Figure 4), the expression of these three inflammatory mediators was readily upregulated following POPC treatment (Figure 7a,b). Similarly, POPC liposomes also induced the expression of IL-6 and IL-10 in J774.1 cells (Figure 7c). Collectively, these data clearly demonstrate that POPC liposomes trigger the expression of numerous inflammatory genes in macrophages, possibly via the activation of the NF-κB pathway.

**Figure 8:**
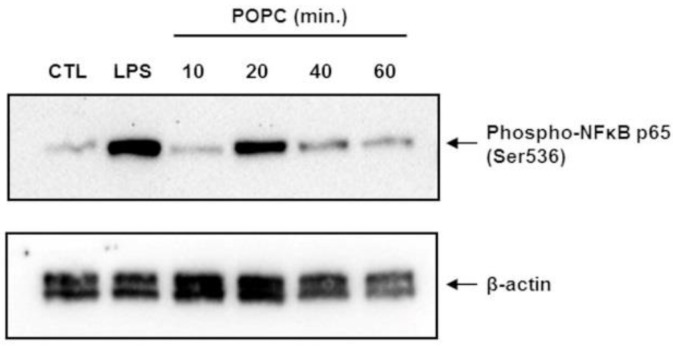
POPC liposome treatment promotes the phosphorylation of the NF-kb p65 subunit. J774A.1 cells were treated with POPC liposomes (2 × 10^5^ per cell) for different periods of time, as indicated. Control cells received PBS vehicle only (no liposome). Cells treated with 100 ng/mL LPS for 10 min were used as positive controls. Cells were lysed in the RIPA lysis buffer containing serine/threonine phosphatases inhibitors, and 60 µg of total protein was resolved by SDS-PAGE. After transfer, Blots were probed with a rabbit anti-phospho-NF-kB p65 mAb followed by HRP-conjugated goat anti-rabbit IgG antibodies. As a loading control, blots were also probed with mouse anti-β-actin monoclonal antibodies followed by goat anti-mouse HRP-conjugated IgG secondary antibodies. Chemiluminescence data were acquired using the ChemiDoc™ MP system. The blot presented is a representative Western blot of multiple experiments (*n* = 3).

To confirm that POPC liposomes indeed activate the NF-κB pathway, we incubated J774A.1 cells with POPC liposomes for various periods of time and assessed the phosphorylation of NF-κB p65 at serine 536 (Ser536) by Western-blot analysis. Control cells were treated with PBS (carrier). J774A.1 cells treated with LPS (100 ng/mL) for 10 min were used as positive controls. We found that POPC liposomes induced a significant increase of NF-κB p65 phosphorylation at Ser536 after 20 min of treatment (Figure 8). These data showing NF-κB activation are in agreement with the increase of TNF-α mRNA expression observed after 30 min of POPC exposure (Figure 6b).

### 3.2. POPC Liposomes Induce Changes in the Proteome of Peritoneal Cells

**Figure 9:**
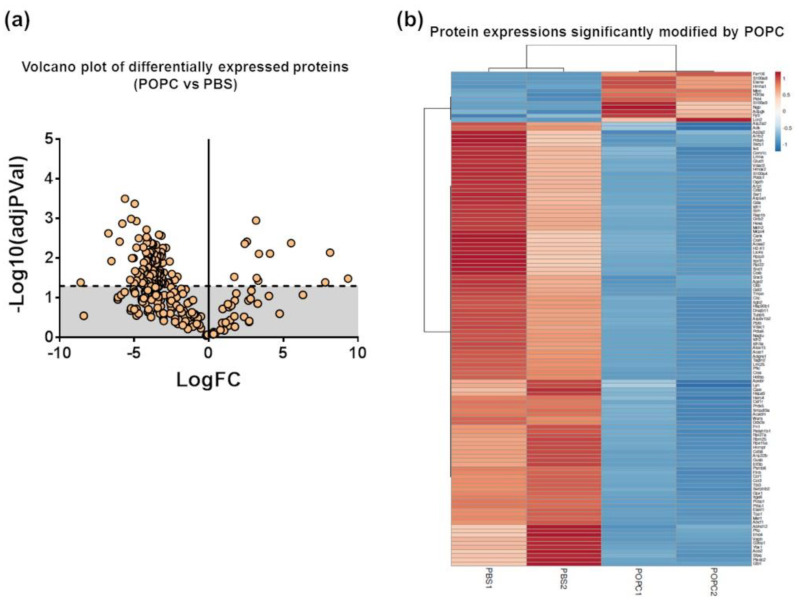

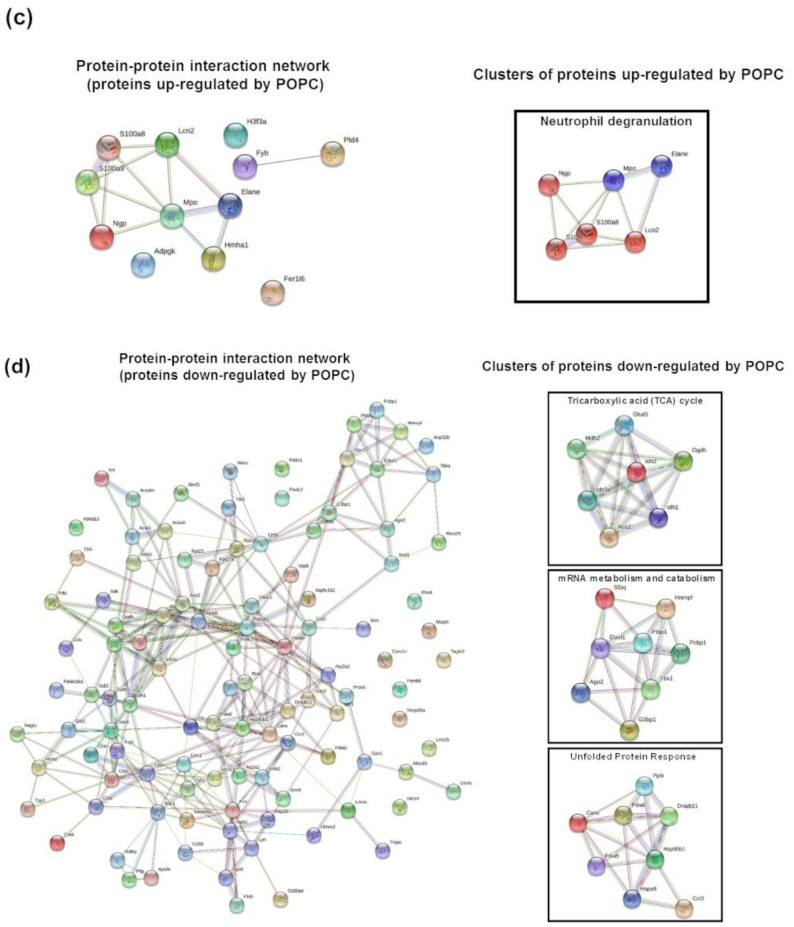
POPC liposomes significantly modify the proteome of peritoneal cells. CD-1 mice were injected in the peritoneal cavity with POPC liposomes (400 µg). Control animals received an equal volume of PBS. Total peritoneal cells were collected 1 h after injection by peritoneal lavage, centrifuged, and cell pellets were analyzed by HPLC coupled with tandem mass spectrometry using nano-spray ionization. MS/MS spectra acquisition, peptide/protein identification, quantification of the data and statistical calculations were performed using the peptide-feature-based PEAKS Studio X. (**a**) Volcano plot showing differentially expressed protein between POPC- and PBS-treated peritoneal cells. (**b**) List of proteins whose expression is significantly modified by POPC treatment. (**c**) Protein–protein interaction network (constructed using the STRING online tool) and a cluster of proteins up-regulated by POPC treatment identified by Reactome analysis. (**d**) Protein–protein interaction network (constructed using the STRING online tool) and a cluster of proteins down-regulated by POPC treatment obtained by Reactome analysis.

We performed a proteomic analysis by mass spectrometry of the same samples used for the transcriptome analysis to complement our data. Peptide sequencing and protein identification were performed using the PEAKS proteomics software, and changes in relative protein abundance between PBS- and POPC-treated peritoneal cells were determined using the PEAKS Q add-on module. A volcano plot showing the differentially expressed proteins between the two groups indicated that the expression of 12 proteins was significantly upregulated after POPC exposure, whereas the expression of 107 proteins was downregulated by this treatment (Figure 9a). The list of differentially expressed proteins is provided as a heatmap displaying the hierarchical clustering obtained by measuring the covariance between replicate samples using Pearson’s correlation coefficient (Figure 9b). To determine functional associations between proteins upregulated by POPC treatment, STRING online tool was used to construct protein–protein interaction (PPI) networks. The PPI network construct indicated that 7 out of the 12 upregulated proteins by POPC treatment were closely associated (Figure 9c), and the Reactome analysis showed that six of these are involved in the neutrophil degranulation pathway (Figure 9c); *p* = 3.16 × 10^−9^ and FDR = 4.74 × 10^−7^). In addition, several clusters of functionally associated proteins were found in the PPI network of proteins downregulated by POPC treatment (Figure 9d). Three highly significant pathways were identified by the Reactome analysis of these protein clusters: The citrate (TCA) cycle pathway (*p* = 1.33 × 10^−15^ and FDR = 3.46 × 10^−14^), the mRNA metabolism/catabolism pathway (*p* = 7.96 × 10^−8^ and FDR = 3.60 × 10^−6^), and the unfolded protein response pathway (UPR; *p* = 2.04 × 10^−13^ and FDR = 1.35 × 10^−11^) (Figure 9d).

### 3.3. POPC Liposomes Elicit the Rapid Recruitment of Neutrophils into the Peritoneal Cavity

**Figure 10:**
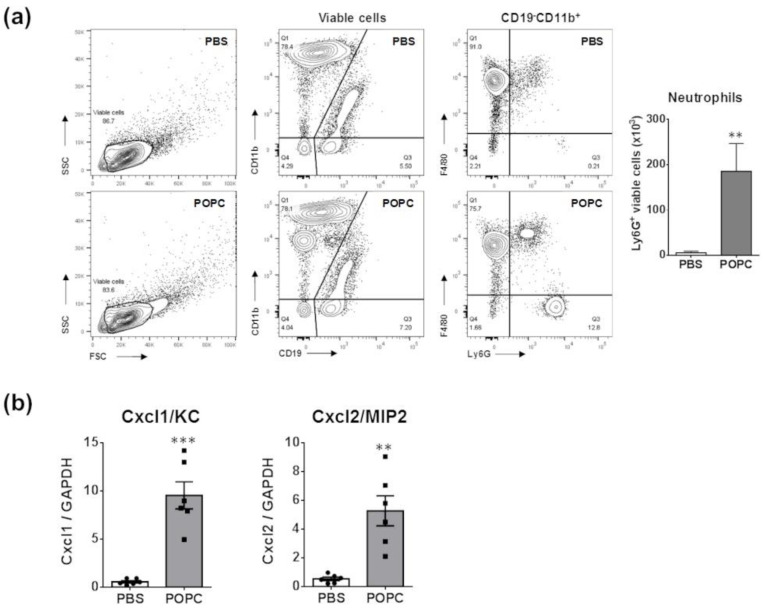
POPC liposomes elicit the rapid recruitment of neutrophils into the peritoneal cavity. (**a**) POPC liposomes (400 µg) or an equal volume of PBS (carrier) were injected into the peritoneal cavity of male CD-1 mice, and peritoneal cells were collected by lavage after 1 h of liposome exposure. The phenotypic analysis of peritoneal cells was performed by flow cytometry using a FACSCanto II flow cytometer as described in the Section 2. The data were analyzed using FlowJo software, and the bar graph indicates the number of viable neutrophils defined as CD19^−^CD11b^+^Ly6G^+^ in POPC-treated vs. PBS-treated peritoneal cells. Data (*n* = 6) are expressed as means±SEM, and statistical analysis for the comparison between groups was performed using unpaired Student’s *t*-test with ** indicating *p* < 0.01 vs. PBS controls. (**b**) CD-1 mice (*n* = 6 per group) were injected with POPC liposomes or an equal volume of PBS (carrier). Peritoneal cells were harvested 1 h after the injection and processed for RNA isolation and qPCR analysis. Bar graphs show the mRNA levels for the neutrophil-recruiting chemokines Cxcl1 and Cxcl2. The housekeeping gene GAPDH was used to normalize data to cDNA inputs. Results are expressed as means ± SEM, and statistical analysis for the comparison between groups was performed using unpaired Student’s *t*-test with ** indicating *p* < 0.01 and *** indicating *p* < 0.001.

An important observation from the proteomic data was the increasing presence of neutrophil granules. Prior studies indicated that neutrophils are not typically found in the peritoneum of naïve mice [11]. Therefore, the presence of these granules may suggest that POPC induces a rapid infiltration of neutrophils into the peritoneum. To test this hypothesis, we injected POPC liposomes (400 µg, corresponding to approximately 2 × 10^12^ liposomes of 100 nm in size) or an equal volume of PBS (carrier) into the peritoneal cavity of male CD-1 mice and collected peritoneal cells by lavage after 1 h of liposome exposure. The phenotypic analysis of peritoneal cells was performed by flow cytometry as previously described [11]. We found that POPC liposomes induced a rapid and limited accumulation of neutrophils (i.e., CD19^−^CD11b^+^F4/80^−^Ly6G^+^ cells) within the peritoneal cavity (Figure 10a). These data suggested that POPC liposomes may stimulate peritoneal macrophages to rapidly release inflammatory mediators involved in the recruitment of neutrophils. In this regard, we observed that the expression of Cxcl1/KC and Cxcl2/MIP2, two chemokines known to favor the recruitment of neutrophils into inflamed tissues, was readily induced in peritoneal cells exposed to POPC liposomes (Figure 10b).

Altogether, these results indicate that PC is capable of initiating an inflammatory response in macrophages by triggering the activation of the NF-κB pathway, which results in the expression of numerous inflammatory mediators, including some involved in the recruitment of neutrophils, such as Cxcl1 and Cxcl2.

## 4. Discussion

The lipid composition of eukaryote cellular membranes is complex, in which glycerophospholipids are the major component, with the additional presence of sphingolipids and sterols [13]. Indeed, the lipid organization of all subcellular membranes, including the plasmalemma, is different and specific for each subcellular compartment [14]. Moreover, phospholipid membranes are key components of the export of vesicles into the extracellular environment, named exosomes or extracellular vesicles, which play a role in intracellular communication [15,16]. Finally, phospholipids are fundamental forming blocks for the assembly of LNP, which has displayed great utility for the delivery of nucleic acids to cells directed at changing gene expression as well as for the development of mRNA vaccines [1,2,3,4,5,6]. The great advantage of LNP is that nucleic acids can be encapsulated at high concentrations into the vesicle lumen and protected from the activity of circulating lytic agents.

Particles containing phospholipid membranes, including LNP, exosomes and apoptotic bodies, are primarily engulfed by phagocytic cells. In this regard, very little attention has been devoted to the possible contribution of phospholipids within these particles to modify cellular functions. The earlier assumption was that phospholipids were inert components [9]. However, other studies have shown that phospholipids could be involved in cellular responses [10]. Certainly, we showed that liposomes made of palmitoyl oleoyl phosphatidylserine (POPS) altered gene expression in macrophages in vivo. Thus, peritoneal injection of POPS liposomes increased the peritoneal expression of cytokines and chemokines that attract neutrophils into the cavity and neutralize bacterial infections [11]. These observations open the possibility that phospholipids are not just bystanders but rather active players in modulating cellular functions.

In the present study, we have expanded our prior investigations on the effect of phospholipids on macrophage function by focusing on palmitoyl oleoyl phosphatidylcholine (POPC) liposomes. Phosphatidylcholine is the most abundant phospholipid in cellular membranes and exosomes. We found that this glycerophospholipid increased the expression of a variety of genes in vivo, particularly those involved in the NF-κB activation pathway, resulting in the expression of cytokines (TNF-α, IL-6 and IL-10) and chemokines (Cxcl1 and Cxcl2). These observations were validated in primary and cultured macrophage lines in ex vivo conditions. We are highly confident that the increase in expression of these inflammatory genes was not due to the endotoxin contamination of POPC liposome preparations since any potential contamination was tested using a very sensitive assay system. In addition, the biological activity of LPS within liposomes is highly reduced due to the entrapment of lipid A, which is the active component of endotoxin, within the lipid bilayer [17]. Since phosphatidylcholine is a scaffold component of the cassette encapsulating the mRNA encoding for the spike protein of the SARS-CoV-2 virus, it is possible that this early activation of innate immune response by phosphatidylcholine may contribute to the success of the COVID-19 vaccine, perhaps acting as an adjuvant.

Exposure of macrophages to POPC liposomes resulted in the specific up-regulation of 146 genes, including *Mfsd2a*, *Tril*, *Tnfsf4* (Table S1), and the down-regulation of 69 genes (e.g., *Epha2*, *Tmem86a*, *Cln8*; Table S2) in comparison with POPS liposomes. Since POPC and POPS contain the same acyl groups, the differences in gene expression modulation by these two phospholipids is likely due to a direct effect of the phospholipid head.

The potential role of phospholipids in modulating cellular physiology is complex due to the independent potential activity of the phospholipid head, acyl groups, or a breakdown of products. We observed that palmitoyl oleoyl phosphatidylglycerol (POPG) or palmitoyl oleoyl phosphatidylethanolamine (POPE) liposomes were harboring the same acyl group as POPC and did not trigger macrophage activation indicated by the absence of TNF-α following stimulation (Figure S1), thus reaffirming the importance of the phospholipid head in the expression of inflammatory genes by PC. In addition, the observation that neither POPG nor POPE, which were prepared under the same conditions as POPC, induced TNF-α expression further discards the possibility that our findings are due to endotoxin contamination. A potential phosphatidylcholine degradation component is lyso-phosphatidylcholine (LysoPC), produced by the activity of phospholipase A1, which could directly activate a cellular signaling pathway [18]. Prior studies have shown that the exogenous addition of LysoPC to immune cells resulted in the expression of inflammatory molecules such as IFN-γ and TNF-α [19,20]. The transcriptome analysis of macrophages exposed to POPC liposomes revealed that the increased expression of the gene encoding for the major facilitator superfamily domain containing 2A (*Mfsd2a*), which is a sodium-dependent LysoPC transporter mainly present on the plasma membrane, but it could also be localized in intracellular membranes such as ER [21]. Mfsd2a transports LysoPC carrying long-chain fatty acids with more than 14-carbon acyl groups. Moreover, the zwitterionic charge of the PC head is critical for Mfsd2a transport [22]. Mfsd2a is upregulated during the activation of CD8^+^ T cells as well as other inflammatory conditions [23,24]. Particularly, its expression could be altered by TNF-α [25]. It is possible that the uptake of POPC liposomes by macrophages increases the expression of Mfsd2a to activate the transport of long-chain fatty acids produced after the degradation of the liposomes from internal vesicular compartments into the cytosol.

Since POPC liposomes are likely to be targeted to the lysosome, a large excess of these vesicles may overload this compartment, triggering lysosomal stress. Lysosomal stress will activate organelle-specific responses, such as biogenesis, exocytosis and autophagy [26,27,28]. Although none of these events were identified within the signal pathways that were modulated by exposure to POPC liposomes, we cannot discard the possibility that an excess of POPC liposomes may result in lysosomal stress. Additionally, phosphatidylcholine could be transferred to other subcellular compartments changing the lipid environment and modulating the activity of resident proteins.

Another gene that was upregulated by incubation with POPC liposomes was *Tril*, which encodes the TLR4 interactor with leucine-rich repeats (Tril). Tril is a modulator of TLR4 signaling, enhancing the activation of the inflammatory cascade [29]. In addition, Tril was also found to modulate the activity of TLR3, which is localized in endosomes [30]. Since POPC liposomes are likely captured by pinocytosis initially accumulating into endosomes, it is possible that the increased expression of Tril may be related to the activation of the innate immune response within this internal compartment.

Finally, a parallel proteomic analysis of the same samples used for the transcriptome analysis showed the alteration of a distinct group of proteins without overlapping with the transcriptome profile. This proteomic analysis revealed the presence of proteins of neutrophil granules within the preparation suggesting the presence of these cells in the peritoneum upon injection of POPC liposomes. This possibility was indeed confirmed by flow cytometry analysis of peritoneal cells after exposure to the liposomes. The presence of neutrophils was small (about 12% of total cells) and transient. The differences in the profile between transcriptomic and proteomic analysis were not surprising since cell functions are regulated at both the transcriptional and translational levels. Thus, the dual transcriptome and proteomic analysis could provide a global view of the response to external agents.

## Figures and Tables

**Table 1:** Signaling Pathway Impact Analysis (SPIA).

Pathway Name	pSize	NDE	pNDE	pGFdr	Status
NF-kappa B signaling pathway	83	29	1.49 × 10^−7^	2.81 × 10^−9^	Activated
Osteoclast differentiation	109	29	6.44 × 10^−5^	4.78 × 10^−7^	Activated
Herpes simplex infection	160	44	3.44 × 10^−7^	2.17 × 10^−6^	Activated
MAPK signaling pathway	199	40	1.92 × 10^−3^	5.99 × 10^−6^	Activated
Cytokine-cytokine receptor interaction	164	34	2.39 × 10^−3^	5.99 × 10^−6^	Activated
Apoptosis	76	24	1.29 × 10^−5^	2.45 × 10^−5^	Activated
Legionellosis	53	21	7.15 × 10^−7^	2.85 × 10^−5^	Activated
Influenza A	137	39	6.05 × 10^−7^	2.85 × 10^−5^	Activated
Tuberculosis	146	27	2.72 × 10^−2^	3.31 × 10^−5^	Activated
Toxoplasmosis	105	32	1.19 × 10^−6^	3.84 × 10^−5^	Activated
Measles	115	34	1.22 × 10^−6^	4.34 × 10^−5^	Activated
NOD-like receptor signaling pathway	54	21	1.03 × 10^−6^	7.28 × 10^−5^	*Inhibited*
Toll-like receptor signaling pathway	85	26	1.09 × 10^−5^	1.64 × 10^−4^	Activated
Jak-STAT signaling pathway	111	30	3.51 × 10^−5^	3.42 × 10^−4^	*Inhibited*
Hepatitis C	96	27	3.99 × 10^−5^	1.05 × 10^−3^	Activated
Malaria	39	14	1.79 × 10^−4^	2.75 × 10^−3^	Activated
Adipocytokine signaling pathway	52	16	4.85 × 10^−4^	3.01 × 10^−3^	Activated
Chagas disease (American trypanosomiasis)	93	23	1.07 × 10^−3^	3.01 × 10^−3^	Activated
Transcriptional misregulation in cancer	134	33	1.09 × 10^−4^	3.01 × 10^−3^	Activated
Chemokine signaling pathway	145	26	4.18 × 10^−2^	3.01 × 10^−3^	Activated
Pertussis	62	17	1.40 × 10^−3^	4.50 × 10^−3^	Activated
Epstein-Barr virus infection	184	41	1.91 × 10^−4^	4.84 × 10^−3^	Activated
RIG-I-like receptor signaling pathway	51	15	1.21 × 10^−3^	1.36 × 10^−2^	Activated
Amyotrophic lateral sclerosis (ALS)	40	8	1.26 × 10^−1^	1.82 × 10^−2^	Activated

**Table 2:** Selected inflammatory genes with increased expression following POPC treatment.

Gene Names	Gene ID	Fold Increase	Adj.*p*-Values
Interleukin-1 alpha	Il1a	37	0.0127
Interleukin-1 beta	Il1b	107	0.0214
Interleukin-6	Il6	44	0.0038
Interleukin-10	Il10	45	0.0155
Tumor necrosis factor-alpha	Tnfa	86	0.0024
Tumor necrosis factor alpha-induced protein 2	Tnfaip2	50	0.0003
C-C motif chemokine 2	Ccl2	66	0.0257
C-C motif chemokine 3	Ccl3	140	0.0184
C-C motif chemokine 4	Ccl4	129	0.0450
C-X-C motif chemokine ligand 1	Cxcl1	118	0.0004
C-X-C motif chemokine ligand 2	Cxcl2	58	0.0004
Colony Stimulating Factor 1	Csf1	14	0.0027
Colony Stimulating Factor 2	Csf2	39	0.0372
Colony Stimulating Factor 3	Csf3	362	0.0313
C-C Motif Chemokine Receptor Like 2	Ccrl2	16	0.0015
Intercellular adhesion molecule 1	Icam1	39	0.0002
Vascular cell adhesion molecule 1	Vcam1	29	0.0201
Toll like receptor 2	Tlr2	17	0.0002
Myeloid differentiation primary response 88	Myd88	4	0.0021

## Data Availability

All the data that support the findings of this study are available in the manuscript, including methods and/or Supplementary Material.

## Supplementary Materials

The following supporting information can be downloaded at: https://www.mdpi.com/article/10.3390/membranes13020141/s1, Table S1: Genes only up-regulated by POPC (*n* = 146); Table S2: Genes only down-regulated by POPC (*n* = 69); Figure S1: Effect of POPC, POPE and POPG liposomes on TNF-α expression.
