# Supplementary material for: Phosphatidylcholine Liposomes Reprogram Macrophages toward an Inflammatory Phenotype
Source: Membranes (Basel). 2023 Jan 21;13(2):141. doi: 10.3390/membranes13020141 (PMC9968183; doi:10.3390/membranes13020141)
Supplement: Supplementary file 1 [file membranes-13-00141-s001.zip › Cauvi et al_Supplemental Figure S1.pdf]

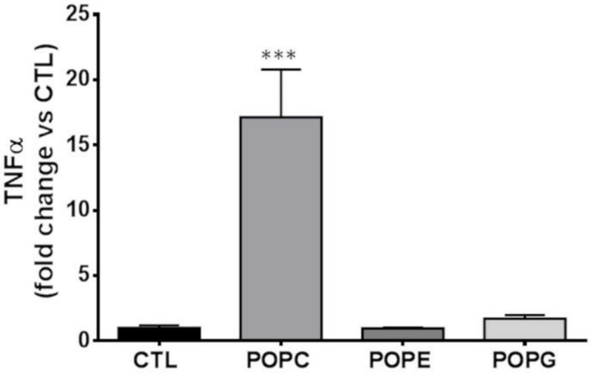


**Figure S1. Effect of POPC, POPE, and POPG liposomes on TNF-α expression.** POPC, POPE, and POPG liposomes were prepared as described in the Materials and Methods section. However, POPE liposome preparation was performed at 30°C, above the phase transition temperature of 1-palmitoyl-2-oleoyl-*sn*-glycero-3-phosphoethanolamine (POPE). J774A.1 cells were treated with POPC, POPE, or POPG liposomes ($2x10^5$ liposomes/cell) for 2 hours, and TNF-α mRNA levels were analyzed by qPCR. The housekeeping gene GAPDH was used to normalize data to cDNA inputs. Results (n=3) are expressed as fold change versus CTL, and statistical analysis was performed using one-way ANOVA ($p<0.001$) followed by Tukey's Multiple Comparison Test with *** indicating $p<0.001$ compared to controls.
